# Multipolar traction with an eight-point adaptive traction device allowed comfortable resection of a challenging giant rectal lesion in ulcerative colitis

**DOI:** 10.1055/a-2155-4772

**Published:** 2023-09-15

**Authors:** Elena De Cristofaro, Jérôme Rivory, Louis-Jean Masgnaux, Jean Grimaldi, Clara Yzet, Sarah Leblanc, Mathieu Pioche

**Affiliations:** 1 Gastroenterology Unit, Department of Systems Medicine, University of Rome Tor Vergata, Rome, Italy; 2 Gastroenterology and Endoscopy Unit, Edouard Herriot Hospital, Hospices Civils de Lyon, Lyon, France; 3 Gastroenterology and Endoscopy Unit, Amiens University Hospital, Amiens, France; 4 Gastroenterology and Endoscopy Unit, Mermoz Hospital, Lyon, France

Current guidelines recommend endoscopic resection for superficial colorectal neoplasia in patients with ulcerative colitis (UC), especially for clearly visible colitis-associated neoplasia 1 . However, endoscopic removal is technically challenging in UC, particularly for giant lesions. The major limitations to the widespread use of endoscopic submucosal dissection are the long procedure duration and the technical difficulty, particularly in the presence of fibrosis 2 . Several devices and techniques have been described to facilitate and speed up the procedure 1 , and traction strategies have been increasingly implemented to improve the efficiency of dissection 3 .

We previously described the benefits of using adaptive traction devices (A-TRACT 2 and A-TRACT 4) to anchor two or four points of the lesion 4 5 . Here, we present the use and benefits of a specially designed adaptive traction device (A-TRACT 8) for multitraction via eight points in a giant rectal lesion in UC.

A 61-year-old man with UC had a large neoplastic area involving three-quarters of the circumference of the rectum. After circumferential incision and trimming, the first two loops were fixed by clips to the oral and anal edges of the target area. Six other loops were fixed on lateral edges of the lesion and another clip was attached to affix the rubber band to the opposite rectal wall ( Fig. 1 , Video 1 ). The dissection was started with appropriate traction. When traction decreased after cutting half of the lesion, we tightened the A-TRACT 8 to re-establish proper traction. Good exposure of the submucosa was achieved thanks to the traction, which facilitated dissection at different depths and under fibrotic areas. The procedure duration was 200 minutes. Complete resection (R0) was achieved, without adverse events. Histopathology revealed low grade dysplasia.

**Fig. 1:**
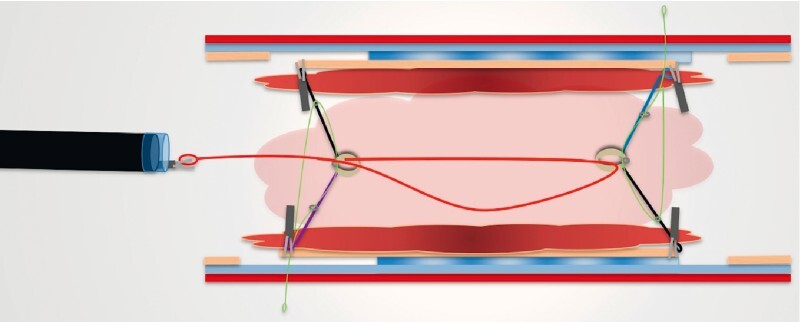
Schematic representation after application of A-TRACT 8, which allowed excellent exposure of the submucosa.

**Video 1** Challenging endoscopic submucosal dissection of a giant rectal lesion facilitated by A-TRACT 8. Image shows rectal dysplasia on ulcerative colitis.


We hypothesize that such a dedicated device could facilitate resection of giant lesions, especially in selected cases, such as patients with inflammatory bowel disease, where the technical difficulties due to fibrosis can represent a great challenge and result in a time-consuming procedure.

Endoscopy_UCTN_Code_TTT_1AQ_2AD

## Notes

Endoscopy E-Videos

https://eref.thieme.de/e-videos
*E-Videos* is an open access online section of the journal *Endoscopy* , reporting on interesting cases and new techniques in gastroenterological endoscopy. All papers include a high-quality video and are published with a Creative Commons CC-BY license. Endoscopy E-Videos qualify for HINARI discounts and waivers and eligibility is automatically checked during the submission process. We grant 100% waivers to articles whose corresponding authors are based in Group A countries and 50% waivers to those who are based in Group B countries as classified by Research4Life (see: https://www.research4life.org/access/eligibility/ ). This section has its own submission website at https://mc.manuscriptcentral.com/e-videos

## References

[1] Bak M TJ Albéniz E East J M Endoscopic management of patients with high-risk colorectal colitis-associated neoplasia: a Delphi study Gastrointest Endosc 2023 97 767 7.79E8 36509111 10.1016/j.gie.2022.12.005

[2] Suzuki N Toyonaga T East J E Endoscopic submucosal dissection of colitis-related dysplasia Endoscopy 2017 49 1237 1242 28806821 10.1055/s-0043-114410

[3] Lafeuille P Rivory J Jacques J Diagnostic endoscopic submucosal dissection for invasive cancer with the four cardinal points traction strategy Endoscopy 2022 54 E281 E282 34215006 10.1055/a-1516-3680

[4] Masgnaux L J Pioche M Rivory J Endoscopic submucosal resection with adaptative traction device: a new strategy to facilitate resection in patient with inflammatory bowel disease Endoscopy 2023 55 01 E466 E467 36828022 10.1055/a-2020-9774 PMC9957667

[5] Grimaldi J Masgnaux L-J Rivory J Multipolar traction with adjustable force increases procedure speed during endoscopic submucosal dissection: the A-TRACT-4 traction device Endoscopy 2022 54 02 E1013 E1014 36002007 10.1055/a-1904-7666 PMC9736797

